# Annotations

**Published:** 1892-12-31

**Authors:** 


### ANNOTATIONS.

"Ring out the Old."

"The year is dying, let him die." It would be better for some of us if a good many other things could die with him. There is no virtue in a date, but there is infinite possibility in a fresh start, if it be really a fresh start. Our dead Tennyson not only bade us "Ring out the old; ring in the new," he bade us also "Watch what main currents draw the years," and "Cut prejudice against the grain." These seem fit ideals for a young and "inspired" generation. One "main current" which is "drawing the years" is the putting off of conventionality, and the putting on of originality; originality being nothing else but a courageous and candid simplicity. We reverence the past, but we judge it too. The best service we can render to the past is to disclaim for ourselves all its inexperience and all its consequent mistakes, and to avoid similar mistakes for ourselves. Thus doing we shall gradually clear the memory of the past, from many things which greatly dim its lustre. The great enemy of prejudice is science; not however always scientific persons. The scientist is as ready as the religionist or the politician to make all manner of abjurations of his own first principles. It is often quite as necessary to "convert" professed scientists to real science, as to insist upon the scientific "new birth" of the great world which lies in unscientific ignorance. If all professed Christians were Christians in practice, it is said the whole world would be Christian in a single generation. Almost without the shade of a change of thought might this be applied to the army and camp followers of science. The first principles, the elements of all great things are few and simple, and they are as true and noble as they are few and simple. "The old" of prejudice, of wilfulness, of self-seeking, is the worthless and the hateful which we are all fain to "ring out." "The old" of first principles, of simplicity, of truth, and of reasonableness is the eternally "new" which every worthy soul is strenuous to "ring in." "The year is dying, let him die," and let everything which is of no worth and injurious die with him. "Ring in the good that is to be."

"Nor Friends, nor Money."

Not everybody will agree with the *Times* that charity is a "fixed" quantity; but all experienced persons know that it is by no means a highly elastic quantity. We do not want to secure for hospitals any more than their proper share; but we do want to obtain for them as much as their proper share. And therefore it is that we constantly ask people to look at the question from a common sense and business point of view. It is clear that money spent on the sick by judicious spenders can hardly do anything but good. Now hospital authorities are judicious spenders, and the sick poor are in such a helpless plight, that all of us who actually see them feel that they really must be helped, even though the robust be allowed to pinch a little in consequence. The healthy may endure hardships; but our feelings of humanity revolt against inflicting starvation and neglect upon the sick. It happens also that the sick in great towns can get exactly what they need at about half the actual value of the work done for them; and this for two reasons: first, because clever doctors are always anxious to secure unpaid hospital appointments; and second, because young women of strong mind and high character are willing to work for very little pay, in order to secure the training of hospital nurses. It comes to this then in practice, that every pound given to hospitals secures two pounds' worth of work; and the work is the most necessitous, and the most benevolent of all work. We cannot, as a nation, allow hospital activity to languish for want of money. We ourselves have, of course, no other than a philanthropic interest in these institutions; and that being so, we feel that we may with just reason claim for hospitals a real precedence among general charities. Since the money of the charitable is, if not a "fixed," at least a very slightly elastic quantity, we urge upon thoughtful readers the necessity for intelligent care in the distribution of their benevolence and alms. The principle of distribution, we venture to suggest, should be this—Provide first for those poor who are in the most serious and immediate straits; and afterwards for the less urgent cases. Now who are in such altogether helpless and terrible straits as those who are sick unto death, and have neither friends nor money?

Trained Almoners.

A suggestion for "Trained Almoners" was the outcome of the Conference on "Co-operation in Charity," held under the presidency of the Bishop of London last week. The suggestion originated with Mr. C. S. Loch, of the Charity Organisation Society. It is good, but it is not new; unless, indeed, Mr. Loch intends that his "trained" almoners shall also be paid agents. In that case the suggestion would be new, but not good. Whilst the grass is growing, the steed is starving; and if the poor are to wait for the "training" of their almoners, a good many of them will die before the training is complete. Besides, we suggest that practical training is the only training which can be of the least possible utility; and practical training can only be attained by actual work among the poor. But is it not the case that hundreds of kind-hearted and thoroughly sensible people are at work already all over the metropolis? These people are excellently trained by long personal experience. It seems to us that the imperatively necessary thing to do is to use the almoners who are already at work; and the one thing needed for their most advantageous employment is to combine them all together into one organisation in every metropolitan district. "Combination" is the real watchword, combination and co-operation. The North Kensington model, to which we have drawn attention on several occasions, is an object lesson, not only for Mr. Loch, but for all unorganised London. This scheme, which combines all existing agencies, has worked successfully for two years. We hope to see it, in its present or some modified form, universally adopted.

M. Pasteur's Seventieth Birthday.

France knows how to honour her distinguished children, and the President of the Republic, with the other notable statesmen and men of light and leading of all professions, who thronged the Sorbonne on Tuesday last to acclaim M. Pasteur on his seventieth birthday, displayed a generous and abundant enthusiasm worthy of a great occasion and a great nation. To English ears the compliments of M. Dupuy, Minister of Public Instruction, seem perhaps a little touched with exaggeration, but even Sir Joseph Lister was compelled by his environment to the use of sounding and eloquent phrase. "There is not in the entire world," said Sir Joseph, addressing M. Pasteur, "a single person to whom medical science is more indebted than to you. . . . Owing to you, surgery has undergone a complete revolution. It has been stripped of its terrors, and its efficiency almost unlimitedly enlarged. . . . You have raised the veil which had for centuries covered infectious diseases." Such is the generous enthusiasm with which one true man of science regards the work of another who is almost a rival. All these things portend something. They portend a new and more kindly era, illuminated by the clear light of science.

